# The Pulse of Various Animals

**Published:** 1874-01

**Authors:** 


### THE PULSE OF VARIOUS ANIMALS.

Vatel, in his "Veterinary Pathology," gives our domestic animals the following pulse : horse, from 32 to 38 pulsations per minute ; ox or cow, 25 to 42 ; ass, 48 to 54 ; sheep, 70 to 79 ; dog, 90 to 100 ; cat, 110 to 120 ; rabbit, 120 ; Guinea-pig, 140 ; duck, 135 ; common fowl, 140.

